# Structure-agnostic protein–ligand binding affinity prediction via hierarchical representation alignment

**DOI:** 10.1093/bioinformatics/btag599

**Published:** 2026-08-10

**Authors:** Xiaowen Hu, Hongyi Huang, Hao Sun, Yunlong Zhao, Zhenggang Wang, Hang Chen, Min Wu, Lei Deng

**Affiliations:** School of Computer Science and Engineering, Central South University, Changsha 410083, China; School of Electrical Engineering & Robotics, Queensland University of Technology, Brisbane, QLD 4000, Australia; School of Computer Science and Engineering, Central South University, Changsha 410083, China; School of Computer Science and Engineering, Central South University, Changsha 410083, China; Hunan Provincial Key Laboratory of Clinical Epidemiology, Xiangya School of Public Health, Central South University, Changsha 410078, China; School of Computer Science and Engineering, Central South University, Changsha 410083, China; Institute for Infocomm Research, Agency for Science, Technology and Research (A*STAR), Singapore 138632, Singapore; School of Computer Science and Engineering, Central South University, Changsha 410083, China

## Abstract

**Motivation:**

To enable real-world protein-ligand affinity prediction, not only out-of-distribution generalization but also robustness to variable structural availability and quality should be considered in model design.

**Results:**

We present AlignNet, a hierarchical representation alignment framework that mitigates intra- and inter-molecular heterogeneity to learn robust protein-ligand embeddings for generalizable affinity prediction, even from sequence-level inputs. Its intra-molecular module projects unimodal and multimodal features into a unified space, aligning augmented multimodal views for feature fusion and unimodal with multimodal embeddings to distill multimodal priors for structure-agnostic inference. Its inter-molecular module aligns protein and ligand embeddings for cross-molecular integration. Extensive experiments show that AlignNet (i) achieves highly competitive performance, with up to a 20.4% gain in SCC on the challenging LBA 30% split under sequence-only settings, suggesting improved out-of-distribution generalization; and (ii) learns well-separated affinity-related clusters, supporting reliable structure-independent prediction.

**Availability and implementation:**

AlignNet is available at https://github.com/altriavin/AlignNet.

## 1 Introduction

Predicting protein–ligand binding affinity is a central task in computational drug discovery, enabling large-scale prioritization of candidate compounds while reducing reliance on costly and time-consuming wet-lab assays (Schirle and Jenkins 2016). Recent advances in deep learning and the growing availability of bioactivity data have made data-driven affinity prediction methods increasingly competitive. A notable line of work focuses on sequence-based models that take protein sequences and ligand SMILES as input (Chen *et al*. 2024). These approaches are appealing because sequence and 1D chemical representations are widely available and support fast inference at scale. However, purely sequence-based models lack explicit structural context, which may hinder further performance improvements, as fine-grained geometric complementarity and local interaction patterns are often pivotal for discriminating subtle binding differences.

To better capture the physical determinants of binding, many contemporary methods incorporate 3D information from proteins, ligands, or their complexes. Structure-based models often achieve improved accuracy by leveraging spatial interaction features. For instance, CheapNet (Lim *et al.* 2025) adopts cross-attention over hierarchical atom–cluster representations to model higher-order molecular interactions. To capitalize on distinct feature views, multimodal approaches integrate sequence and structural signals to learn complementary representations. DataDTA (Zhu *et al*. 2023) aggregates multiscale interaction features from sequences and 3D structures, while MFE (Xu *et al*. 2024) aligns features from protein surfaces, 3D structures, and sequences to obtain a comprehensive protein representation.

Recent advances in self-supervised pretraining have led to large-scale foundation models for proteins and small molecules. Such models span multiple modalities, including sequence-based language models (Lin *et al*. 2023) and 3D structure-based encoders (Zhang *et al*. 2023). We hypothesize that, compared with existing multimodal methods that train sequence and structure encoders from scratch, leveraging modality-specific foundation models as feature extractors can provide transferable priors learned from vast unlabeled corpora, which may benefit downstream tasks under limited labeled data and has been explored for binding affinity prediction (Singh *et al*. 2023). However, effectively combining pretrained models across modalities remains challenging. Embeddings derived from sequence and structure encoders often occupy distinct latent spaces, characterized by discrepancies in feature distributions and inherent biases. This representation mismatch makes naive fusion potentially suboptimal and may hinder the learning of interaction-relevant signals.

Collectively, two aspects appear to remain underexplored in protein–ligand binding affinity prediction. First, since binding affinity arises from the interplay between a protein and a ligand, comprehensive modeling requires bridging the representational heterogeneity found across intra-molecular modalities and inter-molecular entities. Second, while 3D structural information is often highly informative for characterizing protein-ligand interactions, structure-based binding affinity prediction methods that rely on structural inputs at inference time may face practical limitations, particularly when structures are unavailable. Although AlphaFold2 has substantially advanced protein structure prediction, accurately modeling mutant proteins remains challenging (Buel and Walters 2022). Moreover, low-confidence predicted conformations can introduce additional uncertainty into structure-derived features, which may in turn affect the reliability of downstream affinity prediction. These observations motivate a central question: can we exploit structural context during training to enrich representations, while still enabling accurate affinity prediction from sequences alone at inference time?

Based on this observation, we introduce AlignNet, which incorporating intra- and inter-molecular alignment strategies for binding affinity prediction. AlignNet builds upon pretrained encoders for both proteins and ligands, using them as the sequence and structure backbones to extract unimodal representations. The intra-molecular alignment module encodes uni- and multi-modal features with a shared encoder and aligns them in a unified representation space; this not only performs multimodal fusion but also distils multimodal priors into unimodal embeddings, enabling sequence-only inputs to yield informative representations. The inter-molecular alignment module further coordinates protein and ligand embeddings to promote cross-molecular complementarity, producing an interaction-aware fusion representation for the pair. Finally, we introduce an affinity-aware optimization objective that regularizes the embedding geometry so that differences in binding affinity are reflected in distances between embeddings in the latent space, strengthening consistency and generalization. Benchmarking across multiple datasets and interpretability analyses indicate AlignNet’s generalization ability and the reliability of its predictions.

In summary, the main contributions of this paper are as follows: (*i*) We introduce a hierarchical representation alignment strategy that jointly address two key challenges: aligning sequence- and structure-derived embeddings within a unified representation space to alleviate cross-modal heterogeneity, and coordinating protein and ligand representations to enhance cross-molecular compatibility for improved binding affinity prediction. (*ii*) We design a training-inference paradigm that leverages structural information as privileged context during training and distills multimodal priors into unimodal embeddings, enabling accurate affinity prediction with sequence-only inputs when reliable structural data are unavailable. (*iii*) Extensive experiments demonstrate that AlignNet consistently achieves highly competitive performance across challenging cross-dataset, diverse-protein, mutant-protein, and cross-docking benchmarks, with a 20.4% SCC gain on LBA 30% under sequence-only inference.

## 2 Methodology

**Figure 1:**
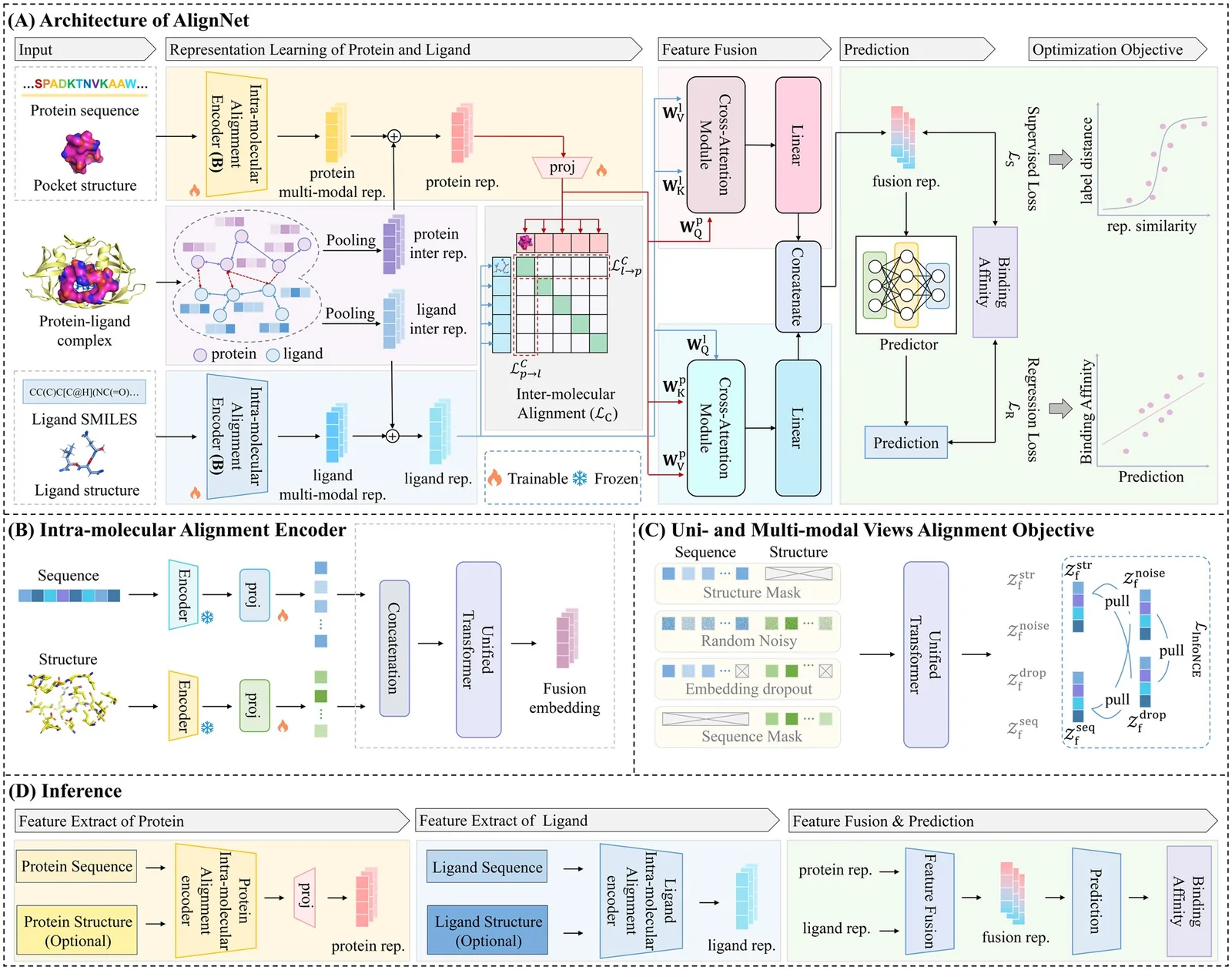
A four-panel diagram illustrating the AlignNet framework for protein-ligand binding affinity prediction. (A) The overall architecture, showing the pipeline from molecular inputs to affinity prediction, including intra- and inter-molecular alignments and optimization objectives. (B) The intra-molecular alignment module, depicting how sequence and structure inputs are encoded, concatenated, and fused via a shared Transformer. (C) The alignment objective, illustrating how four augmented views are aligned in a unified space using an InfoNCE loss. (D) The inference pipeline, demonstrating the affinity prediction process and highlighting its ability to handle sequence-only inputs.

As illustrated in Fig. 1 (pseudo-code provided in Supplementary Section A, available as supplementary data at *Bioinformatics* online), AlignNet is a hierarchical representation alignment architecture that integrates sequence, structure, and protein–ligand complex information to enable robust representation learning and binding affinity prediction under flexible input availability.

### 2.1 Representation learning of protein and ligand

Protein and ligand representation learning proceeds along two paths: the Intra-Molecular Alignment Module yields multimodal embeddings, while the Interaction-Aware Encoding Module derives graph-level embeddings from the protein–ligand complex graph.

#### 2.1.1 Intra-molecular alignment module

To mitigate sequence–structure heterogeneity, we first learn multimodal representations for proteins and ligands separately (Fig. 1B and C). We pretrain the corresponding multimodal encoders on the training set using a uni- and multi-modal alignment objective, and then use them to warm-start end-to-end training for binding affinity prediction. Pseudocode for the Intra-Molecular Alignment module is provided in Supplementary Section B, available as supplementary data at *Bioinformatics* online.

#### 2.1.2 Feature fusion

For a given protein or ligand, we first obtain its sequence embedding $E_{\text{seq}}$ and structure embedding $E_{\text{str}}$ using pretrained encoders. The selection of the pretrained encoder is a hyperparameter (details in Supplementary Section E, available as supplementary data at *Bioinformatics* online). We use ESM-2 (Lin *et al*. 2023) for protein sequences, GearNet (Zhang *et al*. 2023) for protein structures, MolFormer (Ross *et al*. 2022) for ligand sequences, and GraphMVP (Liu *et al*. 2022) for ligand structures. These embeddings are concatenated and passed through a shared Transformer encoder to produce a fused molecular representation $Z_{f}$ for each protein or ligand (Fig. 1B).

#### 2.1.3 Uni- and multi-modal alignment objective

We generate four augmented views of the fused representation, $\left\{Z_{f}^{\text{str}},Z_{f}^{\text{noise}},Z_{f}^{\text{drop}},Z_{f}^{\text{seq}}\right\}$, using structural masking, random noise, embedding dropout, and sequence masking, respectively. The structure- and sequence-masked views are treated as unimodal views, whereas the noise-perturbed and dropout-augmented views preserve multimodal information. The final alignment loss is defined as:


$$\begin{array}{cc} L_{\text{align}}=-I_{\text{NCE}}(Z_{f}^{\text{noise}},Z_{f}^{\text{drop}}) & \\ -\frac{1}{2}\left(I_{\text{NCE}}(Z_{f}^{\text{str}},Z_{f}^{\text{noise}})+I_{\text{NCE}}(Z_{f}^{\text{str}},Z_{f}^{\text{drop}})\right) & \\ -\frac{1}{2}\left(I_{\text{NCE}}(Z_{f}^{\text{seq}},Z_{f}^{\text{noise}})+I_{\text{NCE}}(Z_{f}^{\text{seq}},Z_{f}^{\text{drop}})\right). & \end{array}(1)$$

where $I_{\text{NCE}}(\cdot ,\cdot )$ denotes an InfoNCE-based mutual information objective that pulls together paired views from the same molecule while contrasting them against views from other molecules in the mini-batch (Oord *et al*. 2018). This alignment encourages consistency between multimodal views and transfers multimodal priors to unimodal representations, yielding the aligned protein and ligand representations $Z_{p}^{\text{align}}$ and $Z_{l}^{\text{align}}$, respectively.

#### 2.1.4 Interaction-aware encoding module

We use a geometry-aware graph neural network (GIGN) to model atomic interactions in the protein-ligand complex graph G (Yang *et al*. 2023) and generate graph-level protein and ligand representations ($Z_{p}^{\text{GNN}}$ and $Z_{l}^{\text{GNN}}$; see Supplementary Section C, available as supplementary data at *Bioinformatics* online).

### 2.2 Feature fusion and binding affinity prediction module

The prediction module fuses the aligned and graph-level representations to obtain integrated protein and ligand representations: $Z_{p}={\text{Proj}}_{p}(Z_{p}^{\text{align}}+\delta_{G}Z_{p}^{\text{GNN}}),Z_{l}={\text{Proj}}_{l}(Z_{l}^{\text{align}}+\delta_{G}Z_{l}^{\text{GNN}}),$ where ${\text{Proj}}_{p}(\cdot )$ and ${\text{Proj}}_{l}(\cdot )$ denote linear projections, and $\delta_{G}$ indicates whether the complex graph is available. We then apply cross-attention to obtain context-aware fused representations:


(2) $$\{\begin{array}{c} Z_{p\to l}=\text{softmax}\left((Z_{p}W_{Q}^{p}){\left(Z_{l}W_{K}^{l}\right)}^{T}/\sqrt{d_{k}}\right)(Z_{l}W_{V}^{l}),\\ Z_{l\to p}=\text{softmax}\left((Z_{l}W_{Q}^{l}){\left(Z_{p}W_{K}^{p}\right)}^{T}/\sqrt{d_{k}}\right)(Z_{p}W_{V}^{p}). \end{array}$$

where $W_{Q}^{p},W_{K}^{p},W_{V}^{p}$ and $W_{Q}^{l},W_{K}^{l},W_{V}^{l}$ are learnable query, key, and value projection matrices for the protein and ligand, respectively, and $d_{k}$ denotes the shared key dimension used in both attention directions. The final protein-ligand representation $Z_{\text{pl}}$ is obtained by concatenating the cross-attention outputs: $Z_{\text{pl}}=[Z_{p\to l};Z_{l\to p}],$ which is then passed to an MLP to predict the binding affinity.

### 2.3 Optimization objective

The optimization objective combines three loss functions. The inter-molecular contrastive loss aligns protein and ligand embeddings in a shared representation space, reinforcing the fused protein–ligand embedding. The supervised loss and regression loss serve as an affinity-aware optimization objective, enforcing continuity between the learned protein–ligand features and the binding affinity values.

#### 2.3.1 Inter-molecular contrastive loss ($L_{C}$)

To enforce inter-molecular alignment between interacting molecules, we apply a bidirectional contrastive objective to the integrated protein and ligand representations $Z_{p}$ and $Z_{l}$. For a mini-batch of *N* protein-ligand pairs, the matched protein-ligand pair is treated as the positive sample, while non-matching pairs within the same mini-batch serve as in-batch negatives. This objective encourages the cosine similarity s(·,·) of matched pairs to be higher than that of unmatched pairs:


(3) $$\left\{ \begin{array}{c} L_{p\to l}^{C}(k)=-\text{log}\;\frac{\;\text{exp}\;\left(s(Z_{p,k},Z_{l,k})/\tau \right)}{\sum_{j=1}^{N}\;\text{exp}\;\left(s(Z_{p,k},Z_{l,j})/\tau \right)},\\ L_{l\to p}^{C}(k)=-\text{log}\;\frac{\;\text{exp}\;\left(s(Z_{p,k},Z_{l,k})/\tau \right)}{\sum_{j=1}^{N}\;\text{exp}\;\left(s(Z_{p,j},Z_{l,k})/\tau \right)}, \end{array}\right.$$

where $Z_{p,k}$ and $Z_{l,k}$ denote the integrated representations of the *k*-th protein and ligand in the mini-batch, respectively, τ is a temperature hyperparameter, and s(·,·) denotes cosine similarity. The total inter-molecular contrastive loss is computed by symmetrically averaging the bidirectional losses: $L_{C}=\frac{1}{2N}\sum_{k=1}^{N}\left(L_{p\to l}^{C}(k)+L_{l\to p}^{C}(k)\right)$.

#### 2.3.2 Supervised loss ($L_{S}$)

To better capture the continuous and ordered nature of binding affinity, we incorporate the Rank-n-Contrast (RnC) loss (Zha *et al*. 2023) as the supervised loss. This objective encourages protein-ligand pairs with similar affinity values to be closer in the learned embedding space while preserving affinity-based ranking relationships. For a sample *i*, the set of samples less label-similar to *i* than sample *j* is defined as $S_{i,j}=\{k|k\neq i,d(y_{i},y_{k})\geq d(y_{i},y_{j})\},$ where d(·,·) measures label distance and is defined as $d(y_{i},y_{j})=|y_{i}-y_{j}|$. The supervised loss is defined as:


(4) $$L_{S}=-\frac{1}{N(N-1)}\sum_{i=1}^{N}\sum_{\overset{j=1}{j\neq i}}^{N}\;\text{log}\;\frac{\;\text{exp}\;\left(s(Z_{\text{pl},i},Z_{\text{pl},j})/\tau \right)}{\sum_{k\in S_{i,j}}\;\text{exp}\;\left(s(Z_{\text{pl},i},Z_{\text{pl},k})/\tau \right)}.$$

#### 2.3.3 Regression loss ($L_{R}$)

The regression loss is defined as the Mean Squared Error (MSE) between the predicted and ground-truth binding affinity values in the mini-batch: $L_{R}=\frac{1}{N}\sum_{i=1}^{N}{\left({\hat{y}}_{i}-y_{i}\right)}^{2},$ where ${\hat{y}}_{i}$ and $y_{i}$ denote the predicted and ground-truth affinity values of the *i*-th protein-ligand pair, respectively.

#### 2.3.4 Final loss

The complete loss function for end-to-end training is a combination of the three components: $L=L_{R}+\alpha \cdot L_{S}+\beta \cdot L_{C},$ where α and β are hyperparameters balancing the loss contributions.

### 2.4 Inference

AlignNet accommodates incomplete modalities by aligning uni- and multi-modal representations in a unified space. As shown in Fig. 1D and Algorithm 1, the pretrained encoders extract available molecular features from sequence-only or sequence-and-structure inputs, while graph-level features are used when the protein–ligand complex graph is available. The resulting representations are passed through the fusion and prediction modules to estimate binding affinity.

Algorithm 1 Inference Algorithm of AlignNet **Require**: Optional protein inputs $P_{\text{seq}},P_{\text{struct}}$; optional ligand inputs $L_{\text{seq}},L_{\text{struct}}$; optional interaction graph G. At least one modality is provided for each of the protein and ligand.

**Ensure**: Predicted binding affinity $\hat{y}$

** Stage 1: Intra-Molecular Alignment** 

1: $Z_{p}^{\text{align}}\leftarrow \phi_{\text{fuse}}^{p}({\left\{\phi_{\text{seq}}^{p}(P_{\text{seq}})\right\}}_{P_{\text{seq}}\;\text{avail}},{\left\{\phi_{\text{struct}}^{p}(P_{\text{struct}})\right\}}_{P_{\text{struct}}\;\text{avail}})$

2: $Z_{l}^{\text{align}}\leftarrow \phi_{\text{fuse}}^{l}({\left\{\phi_{\text{seq}}^{l}(L_{\text{seq}})\right\}}_{L_{\text{seq}}\;\text{avail}},{\left\{\phi_{\text{struct}}^{l}(L_{\text{struct}})\right\}}_{L_{\text{struct}}\;\text{avail}})$

** Stage 2: Interaction-Aware Encoding** 

3: **if**  G is provided **then** 

4:  $\left(Z_{p}^{\text{GNN}},Z_{l}^{\text{GNN}})\leftarrow \;\text{InteractionEncodingModule}(G\right)$

5:  $\delta_{G}\leftarrow 1$

6: **else** 

7:  $\left(Z_{p}^{\text{GNN}},Z_{l}^{\text{GNN}})\leftarrow (\prime ,\prime \right)$

8:  $\delta_{G}\leftarrow 0$

9: **end if** 

10: $Z_{p}\leftarrow {\text{Proj}}_{p}(Z_{p}^{\text{align}}+\delta_{G}Z_{p}^{\text{GNN}})$

11: $Z_{l}\leftarrow {\text{Proj}}_{l}(Z_{l}^{\text{align}}+\delta_{G}Z_{l}^{\text{GNN}})$

** Stage 3: Affinity Prediction** 

12: $Z_{p\to l}\leftarrow \text{CrossAttention}(Z_{p},Z_{l})$

13: $Z_{l\to p}\leftarrow \text{CrossAttention}(Z_{l},Z_{p})$

14: $Z_{\text{pl}}\leftarrow \text{Concat}(Z_{p\to l},Z_{l\to p})$

15: $\hat{y}\leftarrow \text{MLP}(Z_{\text{pl}})$

## 3 Experiments

We conduct a comprehensive set of experiments to evaluate AlignNet across multiple challenging scenarios, including Cross-Dataset Generalization, Diverse-Protein Generalization, Mutant-Protein Generalization, Cross-Docking Generalization, and Interpretability Analysis. We compare AlignNet with a broad suite of state-of-the-art (SOTA) baselines, with detailed descriptions provided in Supplementary Section D, available as supplementary data at *Bioinformatics* online. To further understand the contribution of each component, we perform extensive ablation studies in Ablation Study, where we quantify the effects of individual modules and optimization objectives. To further assess the practical utility of AlignNet, we evaluated its performance on the Davis dataset and conducted a large-scale virtual screening experiment, as detailed in Supplementary Sections J and K, available as supplementary data at *Bioinformatics* online, respectively. For robust evaluation, all quantitative results are averaged over three independent runs with different random seeds. Unless otherwise specified, AlignNet is first pretrained via unsupervised Intra-Molecular Alignment using only the training set, which initializes the protein and ligand multimodal encoders before they are fine-tuned for downstream binding affinity prediction. To assess performance under varying input availability, we evaluate three model variants: AlignNet${\;}_{\text{seq}}$ (sequence-only), AlignNet${\;}_{\text{ss}}$ (sequence and structure without complex features), and AlignNet${\;}_{\text{full}}$ (full model). Hyperparameters and implementation details are provided in Supplementary Section E, available as supplementary data at *Bioinformatics* online.

### 3.1 Cross-dataset generalization

Cross-dataset generalization evaluates binding-affinity prediction under distribution shift. Following the data split protocol of GIGN (Yang *et al*. 2023), we train and validate on the PDBbind v2016 general set (11 470 training and 1278 validation samples) and test on the PDBbind v2016 core set (281 samples) and the v2019 holdout set (4314 samples) after filtering out entities that cannot be processed by the pretrained encoders. In this task, we use RMSE and PCC to evaluate model performance. We compare AlignNet against a diverse range of baselines, including sequence-based, structure-based methods and multimodal-based methods, with full implementation details provided in Supplementary Section D, available as supplementary data at *Bioinformatics* online.

Table 1 summarizes the results. The complete results, including standard deviations and statistical analysis, are available in Supplementary Section F, available as supplementary data at *Bioinformatics* online. AlignNet achieves superior overall performance among SOTA baselines. While AlignNet achieves an RMSE on the v2016 core set comparable to the leading baseline, it delivers the most competitive performance on the more challenging v2019 holdout set, improving PCC by 29.8%, even as AlignNet${\;}_{\text{seq}}$ maintains strong predictive power under the sequence-only setting with a 28.0% PCC improvement. This robust performance suggests that AlignNet exhibits stronger generalization ability, enabling accurate predictions on challenging out-of-distribution (OOD) benchmarks.

**Table 1:** Evaluation results of AlignNet and baseline models on the Cross-Dataset Generalization task.^a^

Model	**v2016 set**		**v2019 set**	
	**RMSE** ↓	**PCC** ↑	**RMSE** ↓	**PCC** ↑
**Sequence-based**				
DeepDTA^b^	1.357	0.785	1.485	0.586
GraphDTA^b^	1.434	0.754	1.705	0.474
MGraphDTA^b^	1.439	0.753	1.553	0.538
AttentionSiteDTI^b^	1.352	0.784	1.539	0.563
**Structure-based**				
PotentialNet^b^	1.503	0.772	1.514	0.564
SchNet^b^	1.390	0.787	1.522	0.560
EGNN^b^	1.289	0.816	1.399	0.628
GNN-DTI^b^	1.384	0.779	1.446	0.614
IGN^b^	1.269	0.821	1.410	0.630
GIGN^b^	1.190	0.840	1.393	0.641
CheapNet^b^	**1.107**	0.870	1.343	0.665
**Multimodal based**				
HaPPy^c^	1.213	0.823	–	–
MFE^c^	1.150	0.845	–	–
**Ours**				
AlignNet${}_{\text{seq}}$	1.343	0.969	1.203	0.945
AlignNet${}_{\text{ss}}$	1.264	0.967	1.159	0.947
AlignNet${}_{\text{full}}$	1.213	**0.971**	**1.078**	**0.963**

a The top results are shown in **bold**, and the second-best are underlined, respectively. The dash sign indicates the results of the corresponding methods are not available.

b Results are from CheapNet (Lim *et al.* 2025).

c Results are from MFE (Xu *et al*. 2024).

### 3.2 Diverse-Protein Generalization

Diverse Protein Generalization evaluates binding affinity prediction under stringent protein sequence identity splits. Following the Atom3D protocol (Townshend *et al*. 2021), we split the PDBbind v2019 refined set using protein sequence identity thresholds of 30% (LBA 30%) and 60% (LBA 60%). After filtering entries unsupported by the pretrained encoders, the LBA 30% split contains 3495 training, 466 validation, and 487 test samples, while the LBA 60% split contains 3553 training, 446 validation, and 449 test samples. We evaluate performance using RMSE, PCC, and SCC, and compare AlignNet with SOTA baselines spanning sequence-based, structure-based, multimodal, and pretraining-driven approaches, with full implementation details provided in Supplementary Section D, available as supplementary data at *Bioinformatics* online.

As shown in Table 2 (with complete results, including standard deviations and statistical analyses, provided in Supplementary Section F, available as supplementary data at *Bioinformatics* online), AlignNet${\;}_{\text{full}}$ achieves the best performance among the SOTA baselines. On the LBA 60% split, it improves PCC and SCC by 12.8% and 16.7%, respectively, over the strongest baseline. On the more challenging LBA 30% split, AlignNet${\;}_{\text{full}}$ achieves gains of 22.6% in PCC and 24.2% in SCC. Under the sequence-only input setting, AlignNet${\;}_{\text{seq}}$ remains competitive, improving PCC and SCC by 18.5% and 20.4% on LBA 30%, and by 12.5% and 16.3% on LBA 60%, respectively. Together with the results on the v2019 set, these findings demonstrate that AlignNet achieves stronger OOD generalization than existing SOTA baselines, particularly under challenging protein-level distribution shifts.

**Table 2:** Evaluation results of AlignNet and baseline models on the Diverse-Protein Generalization task.^a^

Model	**LBA 30%**			**LBA 60%**		
	**RMSE** ↓	**PCC** ↑	**SCC** ↑	**RMSE** ↓	**PCC** ↑	**SCC** ↑
**Sequence-based**						
DeepDTA^b^	1.866	0.472	0.471	1.762	0.666	0.663
SSA^b^	1.985	0.165	0.152	1.891	0.249	0.275
TAPE^b^	1.890	0.338	0.286	1.633	0.568	0.571
**Structure-based**						
Atom3D-GNN^b^	1.601	0.545	0.533	1.408	0.743	0.743
IEConv^b^	1.554	0.414	0.428	1.473	0.667	0.675
ProNet^b^	1.463	0.551	0.551	1.343	0.765	0.761
SchNet^b^	1.370	0.590	0.571	–	–	–
LEFTNet^b^	1.366	0.592	0.580	–	–	–
GET^b^	1.327	0.620	0.611	–	–	–
CheapNet^b^	1.311	0.642	0.639	1.238	0.794	0.789
**Pretraining-based**						
EGNN-PLM^b^	1.403	0.565	0.544	1.559	0.644	0.646
ProFSA^b^	1.377	0.628	0.620	1.377	0.764	0.762
**Multimodal-based**						
HaPPy^c^	1.814	0.612	0.606	2.179	0.699	0.698
MFE^c^	1.941	0.598	0.594	1.813	0.759	0.763
**Ours**						
AlignNet${}_{\text{seq}}$	1.464	0.827	0.843	1.221	0.919	0.952
AlignNet${}_{\text{ss}}$	1.465	0.846	0.847	1.227	0.921	0.952
AlignNet${}_{\text{full}}$	**1.301**	**0.868**	**0.881**	**1.056**	**0.922**	**0.956**

a The top results are shown in **bold**, and the second-best are underlined, respectively. The dash sign indicates the results of the corresponding methods are not available.

b Results are from CheapNet (Lim *et al.* 2025).

c Results are from source code.

### 3.3 Mutant-protein generalization

Mutations at or near pocket can perturb local protein conformation and dynamics, substantially altering ligand-binding affinity and making mutant-protein generalization challenging (Duan *et al*. 2023). To evaluate AlignNet in this mutant-protein setting, we use the Platinum database (Pires *et al*. 2015), which provides experimentally measured protein–ligand binding affinities and mutation-induced structural changes. To ensure fair comparison with structure-based baselines and avoid uncertainty from predicted mutant structures (Buel and Walters 2022), we retain only entries with experimentally determined mutant protein–ligand complexes. After removing entries whose proteins overlap with the training set, the final benchmark contains 134 samples, with details provided in Supplementary Section G, available as supplementary data at *Bioinformatics* online. We report PCC and SCC, and compare AlignNet with sequence-based, structure-based, pretraining-based, and multimodal methods; baseline details are provided in Supplementary Section D, available as supplementary data at *Bioinformatics* online.

**Figure 2:**
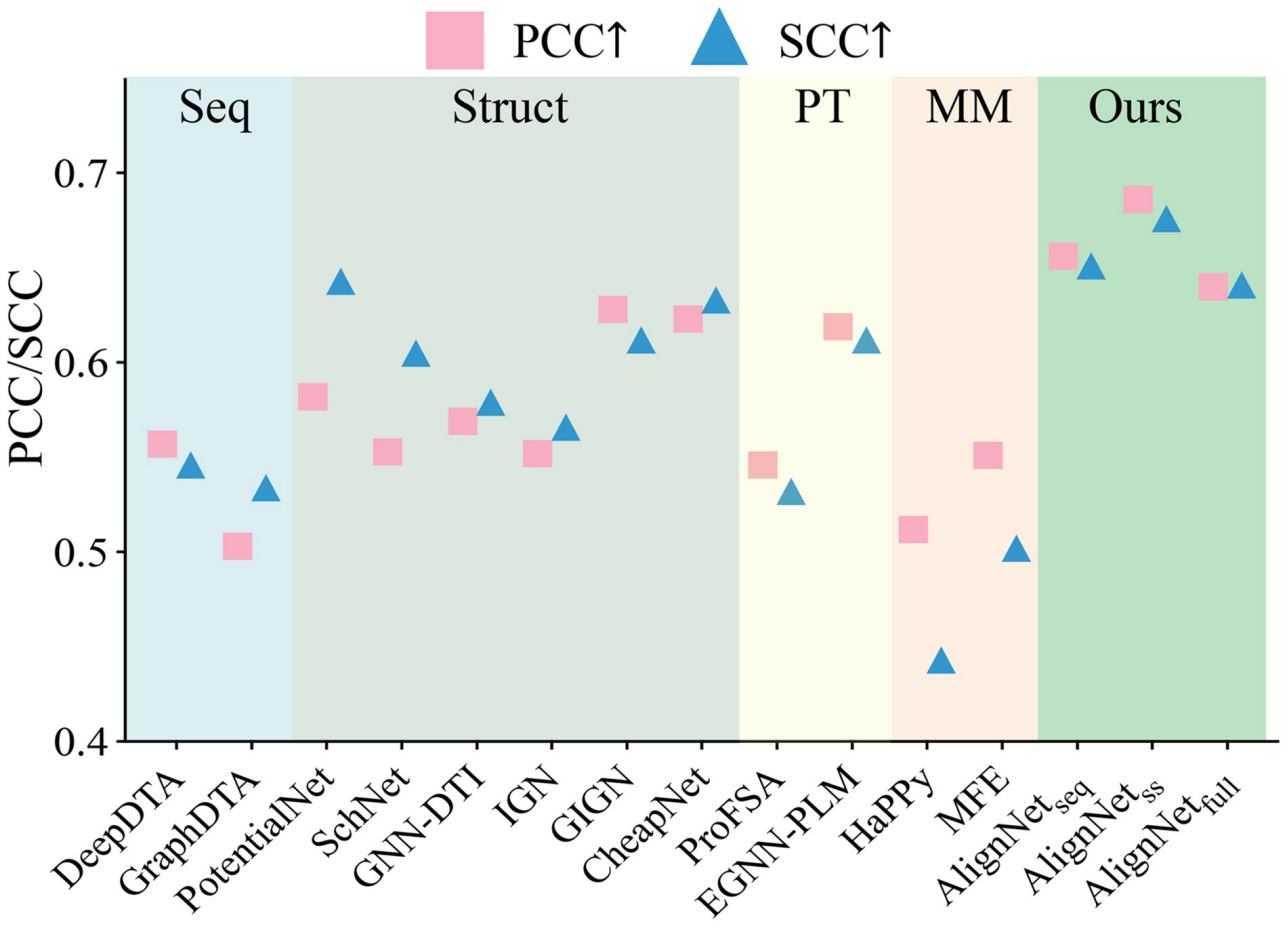
Performance comparison of AlignNet and baseline models on the Mutant-Protein Generalization task. Our methods (Ours) are compared against sequence-based (Seq), structure-based (Struct), pretraining-based (PT), and multimodal-based (MM) approaches on the Platinum dataset. Baseline results are derived from their official source codes.

Figure 2 shows that AlignNet${\;}_{\text{ss}}$ attains the strongest overall performance on Platinum, achieving a PCC of 0.686 and an SCC of 0.676. Compared with the strongest competing baselines, this corresponds to an improvement of 5.8% in PCC and 3.3% in SCC. Notably, AlignNet${\;}_{\text{seq}}$ yields the next-best results (PCC 0.656, SCC 0.651), suggesting that the proposed training strategy can endow sequence-only inputs with informative representations and competitive accuracy, which is particularly valuable in settings where reliable mutant structures are unavailable. However, we observe that AlignNet${\;}_{\text{full}}$ does not further improve over AlignNet${\;}_{\text{ss}}$ on Platinum. One plausible explanation is that the additional structure-derived features may not always be aligned with the determinants of affinity change, such as mutations that occur outside the binding pocket may introduce subtle global or dynamic effects that are difficult to capture from pocket-ligand graph. In such cases, incorporating complex-level structural features could introduce noise or bias the model toward local interaction patterns, potentially reducing robustness.

### 3.4 Cross-Docking Generalization

Cross-Docking Generalization tests model robustness under realistic receptor-conformation mismatch by pairing ligands with non-cognate receptor structures. Following GAABind (Tan *et al*. 2024), we evaluate AlignNet on the COVID Moonshot dataset (Saar *et al*. 2023), which contains crystallographic complexes of SARS-CoV-2 main protease with diverse ligands. The construction details of the COVID Moonshot dataset are provided in Supplementary Section H, available as supplementary data at *Bioinformatics* online. After excluding entries without reported binding affinities, the final dataset contains 119 cross-docked complexes. We report PCC and SCC, and compare AlignNet with SOTA methods, including complex-based and complex-free approaches; details are provided in Supplementary Section D, available as supplementary data at *Bioinformatics* online.

**Figure 3:**
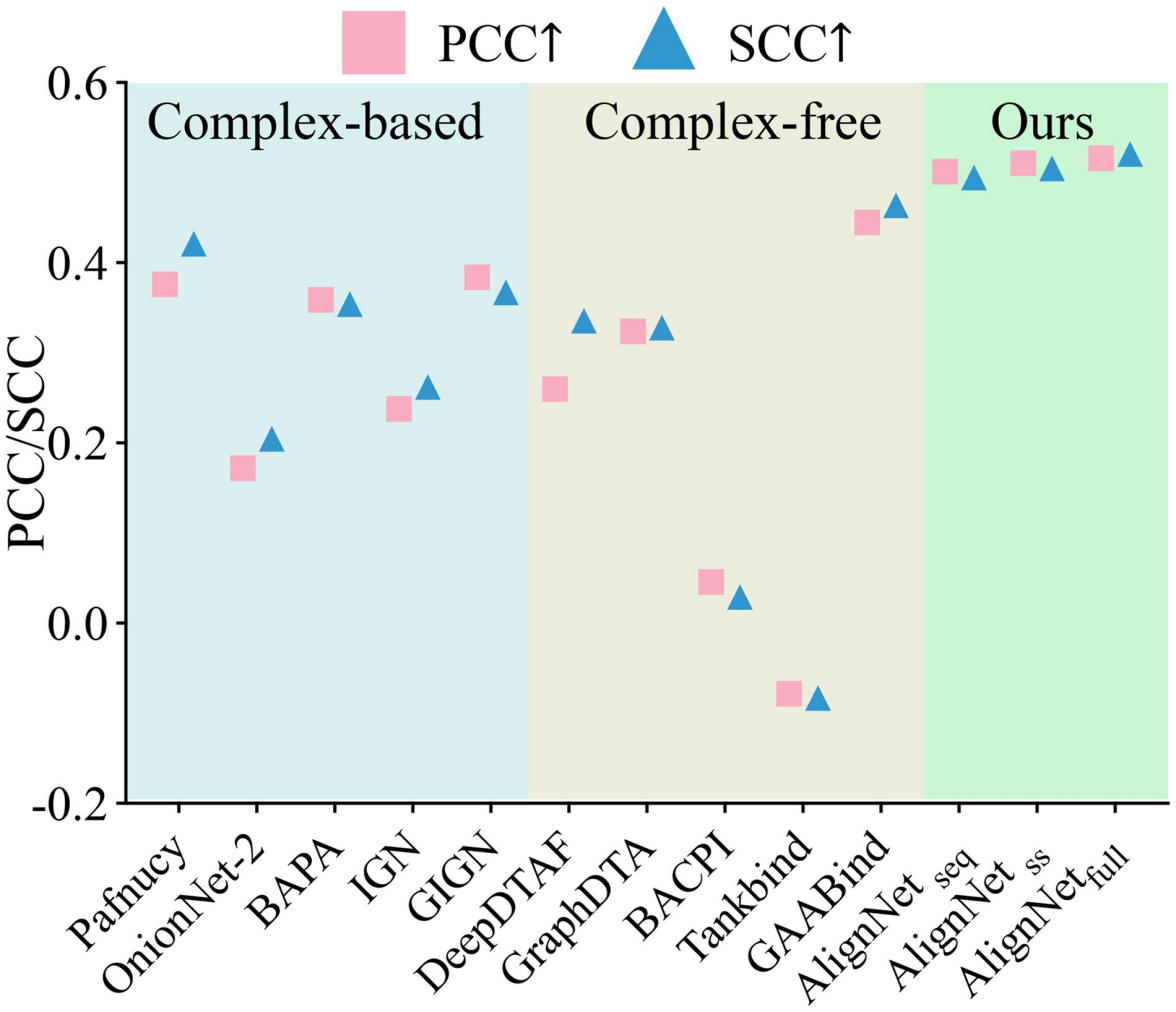
Performance comparison of AlignNet and baseline methods on the COVID Moonshot dataset under the Cross-Docking Generalization setting. The baseline results are adapted from the GAABind (Tan *et al*. 2024).

As summarized in Fig. 3, AlignNet${\;}_{\text{full}}$ attains the best performance (PCC 0.516, SCC 0.521), with relative improvement of 7.1% in PCC and 5.7% in SCC over the strongest baseline GAABind. Relative to the best complex-based baselines, the improvements are 13.2% and 10.0%, respectively. The complex-free AlignNet${\;}_{\text{ss}}$ also surpasses all baselines, improving over GAABind by 6.6% (PCC) and 4.1% (SCC), and over the best complex-based methods by +12.7% (PCC) and +8.4% (SCC). Notably, even AlignNet${\;}_{\text{seq}}$ exceeds every baseline, with 5.6% (PCC) and 3.1% (SCC) improvement over GAABind, underscoring robustness to protein conformational variability.

### 3.5 Interpretability analysis

**Figure 4:**
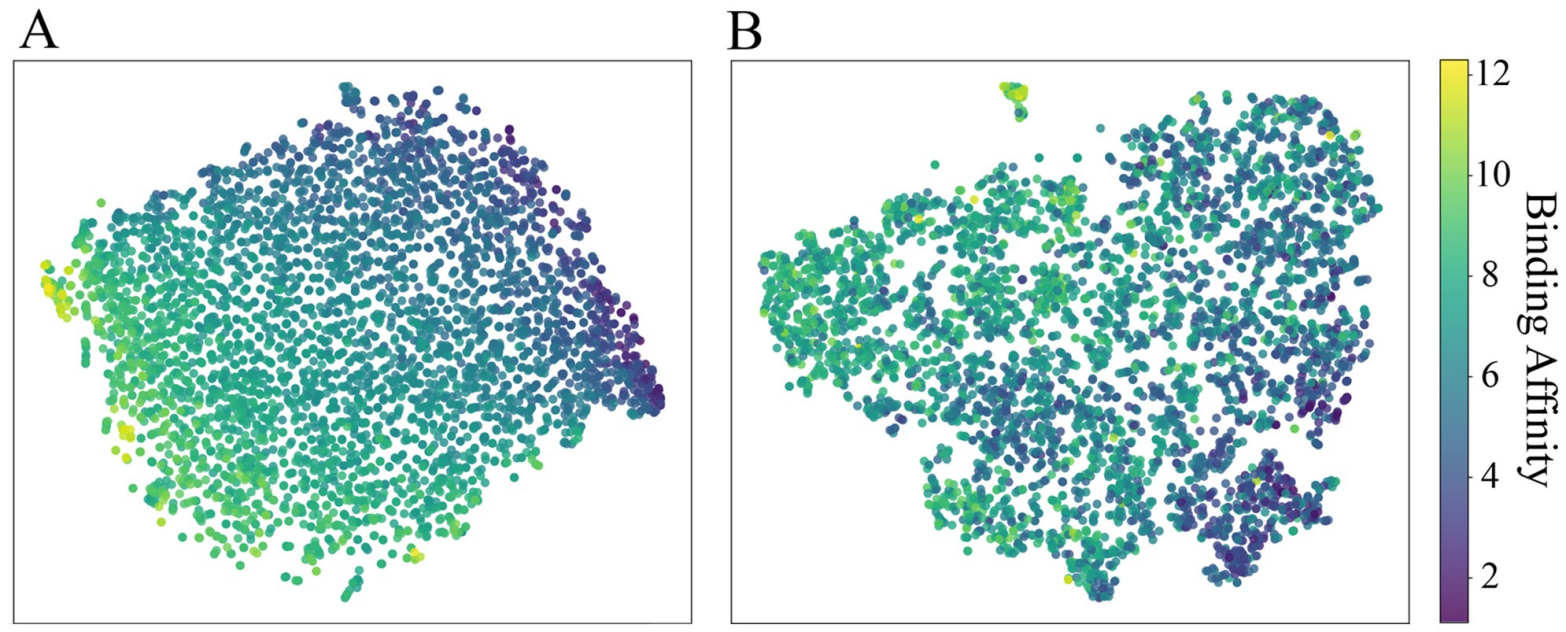
t-SNE visualizations of the final complex embeddings on the v2019 holdout set, colored by binding affinity. Panels (A) and (B) show the embedding spaces learned by AlignNet and CheapNet, respectively.

To qualitatively interpret the learned representations, we visualized the final fused protein-ligand embeddings ($Z_{\text{pl}}$) using t-SNE. Figure 4 shows the two-dimensional projections of the v2019 holdout set, with each sample colored according to its ground-truth binding affinity. For comparison, we visualized the embeddings produced by AlignNet${\;}_{\text{full}}$ (Fig. 4A) and a strong baseline, CheapNet (Fig. 4B). The embeddings learned by AlignNet form a coherent and smoothly varying manifold, where samples with similar binding affinities tend to be located close to each other and the color distribution changes gradually across the embedding space. This strong consistency between the embedding geometry and binding affinity suggests that AlignNet learns affinity-aware representations rather than task-irrelevant features. In contrast, the embeddings produced by CheapNet are more scattered and show weaker global organization with respect to binding affinity. These observations provide qualitative evidence that the fused representations learned by AlignNet are more interpretable, as their structure in the embedding space reflects the underlying binding affinity relationships.

### 3.6 Ablation study

#### 3.6.1 Ablation on model components

We compare six ablated variants: *w/o PF* and *w/o LF*, which remove protein and ligand multimodal fusion, respectively; *w/o BF*, which removes both; and *w/o IG*, which discards features from the complex interaction graph. In addition, *w/o UM* and *w/o MM* remove uni-modal augmented views (structure and sequence masking) and multimodal augmented views (random noise and embedding dropout), respectively, within the Intra-Molecular Alignment module (Fig. 1C). As shown in Table 3, both uni-modal and multimodal augmentations contribute to the best performance. These results suggest that pretrained encoders provide a strong initialization, while the proposed alignment strategy further adapts pretrained embeddings into task-relevant protein-ligand representations. Moreover, although the interaction graph contributes to overall performance gains, removing alignment for either the protein or ligand leads to a clear performance decline, and removing both results in the largest drop.

**Table 3:** Ablation study on the architectural components of AlignNet across LBA 30% and LBA 60% dataset.

	**LBA 30%**			**LBA 60%**		
	**RMSE** ↓	**PCC** ↑	**SCC** ↑	**RMSE** ↓	**PCC** ↑	**SCC** ↑
AlignNet ${}_{\text{Full}}$	**1.301**	**0.868**	0.881	**1.056**	0.922	**0.956**
*w/o PF*	1.471	0.861	0.850	1.747	**0.929**	0.943
*w/o LF*	1.669	0.852	0.857	1.843	0.919	0.938
*w/o BF*	1.737	0.839	0.849	1.846	0.908	0.933
*w/o IG*	1.442	0.845	**0.882**	1.588	0.913	0.938
*w/o UM*	1.501	0.804	0.851	1.756	0.889	0.933
*w/o MM*	1.868	0.768	0.771	1.857	0.891	0.929

#### 3.6.2 Ablation on loss functions

We ablate the three loss-terms in AlignNet’s objective: the regression loss ($L_{R}$), the inter-molecular contrastive loss ($L_{C}$), and the supervised loss ($L_{S}$). As shown in Table 4, removing $L_{R}$ leads to severe performance degradation, underscoring the importance of the MSE loss for fundamental label fitting. Building on this foundation, incorporating either $L_{C}$ or $L_{S}$ leads to notable performance improvements, indicating that both the inter-molecular module and the supervised loss provide beneficial training signals. Finally, combining all three loss functions yields the most competitive overall performance, demonstrating the effectiveness of the proposed joint optimization strategy.

**Table 4:** Ablation study on the loss function across LBA 30% and LBA 60% dataset.

Loss Components			LBA 30%			LBA 60%		
$L_{R}$	$L_{S}$	$L_{C}$	RMSE ↓	PCC ↑	SCC ↑	RMSE ↓	PCC ↑	SCC ↑
**✓**	**✓**	**✓**	**1.301**	**0.868**	**0.881**	**1.056**	**0.922**	**0.956**
✓	✓		1.377	0.848	0.860	1.550	0.901	0.928
✓		✓	1.627	0.795	0.825	1.606	0.890	0.924
✓			1.725	0.591	0.542	2.144	0.608	0.654
	✓	✓	6.440	0.609	0.627	6.755	0.686	0.672
	✓		6.442	0.517	0.506	6.552	0.421	0.405
		✓	6.185	0.054	−0.002	6.425	−0.010	0.002

#### 3.6.3 Ablation of the Intra-Molecular Alignment module

To investigate the contribution of the structural knowledge distillation process, we introduce a control variant, AlignNet${\;}_{\text{se}}$. This baseline is trained directly from the pre-trained sequence encoders, bypassing the Intra-Molecular Alignment module. The results on the LBA 30% and LBA 60% datasets are presented in Table 5. As observed, AlignNet${\;}_{\text{seq}}$ yields improved performance over the AlignNet${\;}_{\text{se}}$ baseline across all metrics. It is worth noting that both models rely exclusively on sequence-only inputs during the inference phase. Therefore, this performance difference suggests that the Intra-Molecular Alignment module effectively helps distill structural priors into the unimodal sequence embeddings during training, which allows for more accurate affinity predictions when structural data are unavailable.

**Table 5:** Ablation study on the Intra-Molecular Alignment Module of AlignNet across LBA 30% and LBA 60% dataset.

	**LBA 30%**			**LBA 60%**		
	**RMSE** ↓	**PCC** ↑	**SCC** ↑	**RMSE** ↓	**PCC** ↑	**SCC** ↑
AlignNet ${}_{\text{se}}$	1.600	0.813	0.824	1.950	0.892	0.922
AlignNet ${}_{\text{seq}}$	**1.464**	**0.827**	**0.843**	**1.221**	**0.919**	**0.952**

#### 3.6.4 Computational efficiency

All models were implemented in PyTorch and trained on an Ubuntu server equipped with 256GB of RAM and NVIDIA RTX 4090 (24GB) and NVIDIA RTX A6000 (48GB) GPUs. The detailed computational efficiency of AlignNet in the pretraining, training and inference, as shown in Table 6.

**Table 6:** Computational efficiency of AlignNet.

Stage	Peak GPU Mem (MiB)	Wall-clock	Epochs
Pretraining (protein)	44 724	16 h	100
Pretraining (ligand)	20 838	3 h	100
Training	18 187	1.5 h	30
Inference	4897	∼25 s	–

*Note.* We first use the pretrained encoders to compute per-protein and per-ligand embeddings; this one-off preprocessing takes ∼2 h and ∼55 GB of storage.

## 4 Conclusion

In this study, we present AlignNet, a representation learning framework that bridges structure-based training and sequence-based inference for protein–ligand binding affinity prediction. AlignNet aligns sequence- and structure-derived representations within a unified latent space to reduce cross-modal heterogeneity, while coordinating protein and ligand representations to enhance cross-molecular compatibility. Extensive experiments demonstrate that AlignNet achieves strong generalization and robustness under diverse out-of-distribution settings, particularly in sequence-only scenarios where structural information is unavailable or unreliable. Compared with existing approaches, AlignNet uniquely balances representation expressiveness and predictive flexibility, with interpretability analyses supporting the reliability of its structure-independent inference. By reducing reliance on high-quality structural inputs, AlignNet has the potential to streamline target-based drug discovery workflows and mitigate trial-and-error in early-stage screening. More broadly, we anticipate that the proposed alignment-based strategy will inspire future work on structure-free and multimodal molecular representation learning across related biological interaction modeling. Although our method achieves impressive performance, some limitations should still be noted. While our model uses sequence-only input during inference, structural data are still required during training. Therefore, exploring large-scale pretraining strategies that enable both fine-tuning and inference using sequence-only input remains an important direction for future work.

## Supplementary Material

btag599_Supplementary_Data

## Data Availability

The source code and dataset are available at https://github.com/altriavin/AlignNet.
