# Supplementary material for: Structure-agnostic protein–ligand binding affinity prediction via hierarchical representation alignment
Source: Bioinformatics. 2026 Aug 10;42(8):btag599. doi: 10.1093/bioinformatics/btag599 (PMC13493050; doi:10.1093/bioinformatics/btag599)
Supplement: Supplementary file 1 [file btag599_supplementary_data.pdf]

Supplementary Materials for Structure-Agnostic Protein–Ligand Binding Affinity Prediction via Hierarchical Representation Alignment

Xiaowen Hu[1], Hongyi Huang[2], Hao Sun[1], Yunlong Zhao[1], Zhenggang Wang[3], Hang Chen[1], Min Wu[4], and Lei Deng[1]∗

[1] School of Computer Science and Engineering, Central South University, Changsha, 410083, China
[2] School of Electrical Engineering & Robotics, Queensland University of Technology, Brisbane, Australia, 4000
[3] Hunan Provincial Key Laboratory of Clinical Epidemiology, Xiangya School of Public Health, Central South University, Changsha, 410078, China
[4] Institute for Infocomm Research, Agency for Science, Technology and Research (A*STAR), Singapore, 138632
∗ Corresponding author
Correspondence to `leideng@csu.edu.cn`

A Pseudo-Code of AlignNet

In this section, we provide the detailed pseudo-code for the end-to-end training process of AlignNet (Algorithm 1). The intra-molecular alignment encoders are first pretrained using Algorithm 2 and then used to warm-start the binding affinity prediction model.

Algorithm 1 AlignNet: End-to-End Training Algorithm

Require: A mini-batch of protein–ligand data $\mathcal{B} = \{(P_{i,\text{seq}}, P_{i,\text{str}}, L_{i,\text{seq}}, L_{i,\text{str}}, \mathcal{G}_i, \delta_{\mathcal{G}_i}, y_i)\}_{i=1}^{N}$
Require: Warm-started intra-molecular alignment encoder Φ_{align} from Algorithm 2
Require: GIGN encoder Φ_{GNN}, projection layers $\text{Proj}_p(\cdot)$ and $\text{Proj}_l(\cdot)$
Require: Cross-attention parameters $\{\mathcal{W}_Q^p, \mathcal{W}_K^p, \mathcal{W}_V^p, \mathcal{W}_Q^l, \mathcal{W}_K^l, \mathcal{W}_V^l\}$, prediction head $\text{MLP}(\cdot)$
Require: Loss weights α, β, temperature τ
Ensure: Total loss $\mathcal{L}$ and predicted binding affinities $\{\hat{y}_i\}_{i=1}^{N}$

Initialize $\mathcal{Z}_p \leftarrow []$, $\mathcal{Z}_l \leftarrow []$, $\mathcal{Z}_{\text{pl}} \leftarrow []$, $\hat{\mathcal{Y}} \leftarrow []$
for each protein–ligand pair $(P_i, L_i, \mathcal{G}_i, y_i)$ in $\mathcal{B}$ **do**
  Stage 1: Intra-molecular aligned representation learning
  $\mathcal{Z}_{p,i}^{\text{align}} \leftarrow \Phi_{\text{align}}(P_{i,\text{seq}}, P_{i,\text{str}})$
  $\mathcal{Z}_{l,i}^{\text{align}} \leftarrow \Phi_{\text{align}}(L_{i,\text{seq}}, L_{i,\text{str}})$
  Stage 2: Interaction-aware graph encoding
  if $\delta_{\mathcal{G}_i} = 1$ **then**
    $(\mathcal{Z}_{p,i}^{\text{GNN}}, \mathcal{Z}_{l,i}^{\text{GNN}}) \leftarrow \text{GIGN}(\mathcal{G}_i; \Phi_{\text{GNN}})$
  else
    $\mathcal{Z}_{p,i}^{\text{GNN}} \leftarrow \mathbf{0}$, $\mathcal{Z}_{l,i}^{\text{GNN}} \leftarrow \mathbf{0}$
  end if
  Stage 3: Feature fusion and affinity prediction
  $\mathcal{Z}_{p,i} \leftarrow \text{Proj}_p\left(\mathcal{Z}_{p,i}^{\text{align}} + \delta_{\mathcal{G}_i}\mathcal{Z}_{p,i}^{\text{GNN}}\right)$
  $\mathcal{Z}_{l,i} \leftarrow \text{Proj}_l\left(\mathcal{Z}_{l,i}^{\text{align}} + \delta_{\mathcal{G}_i}\mathcal{Z}_{l,i}^{\text{GNN}}\right)$
  $\mathcal{Z}_{p\to l,i} \leftarrow \text{CrossAttention}_{p\to l}\left(\mathcal{Z}_{p,i}, \mathcal{Z}_{l,i}; \mathcal{W}_Q^p, \mathcal{W}_K^l, \mathcal{W}_V^l\right)$
  $\mathcal{Z}_{l\to p,i} \leftarrow \text{CrossAttention}_{l\to p}\left(\mathcal{Z}_{l,i}, \mathcal{Z}_{p,i}; \mathcal{W}_Q^l, \mathcal{W}_K^p, \mathcal{W}_V^p\right)$
  $\mathcal{Z}_{\text{pl},i} \leftarrow [\mathcal{Z}_{p\to l,i}; \mathcal{Z}_{l\to p,i}]$
  $\hat{y}_i \leftarrow \text{MLP}(\mathcal{Z}_{\text{pl},i})$
  Append $\mathcal{Z}_{p,i}$ to $\mathcal{Z}_p$, $\mathcal{Z}_{l,i}$ to $\mathcal{Z}_l$
  Append $\mathcal{Z}_{\text{pl},i}$ to $\mathcal{Z}_{\text{pl}}$, $\hat{y}_i$ to $\hat{\mathcal{Y}}$
end for
Training objective over the mini-batch
$\mathcal{L}_{\text{C}} \leftarrow \text{InterMolecularContrastiveLoss}(\mathcal{Z}_p, \mathcal{Z}_l; \tau)$
$\mathcal{L}_{\text{S}} \leftarrow \text{RankNContrastLoss}(\mathcal{Z}_{\text{pl}}, \{y_i\}_{i=1}^{N}; \tau)$
$\mathcal{L}_{\text{R}} \leftarrow \frac{1}{N}\sum_{i=1}^{N}(\hat{y}_i - y_i)^2$
$\mathcal{L} \leftarrow \mathcal{L}_{\text{R}} + \alpha\mathcal{L}_{\text{S}} + \beta\mathcal{L}_{\text{C}}$
Update model parameters by back-propagating $\mathcal{L}$

B Pseudo-Code of Intra-Molecular Alignment Module

In this section, we provide the detailed pseudo-code for pretraining the Intra-Molecular Alignment Module (Algorithm 2). The learned encoder is used to produce the aligned protein and ligand representations $\mathcal{Z}_p^{\text{align}}$ and $\mathcal{Z}_l^{\text{align}}$ for end-to-end AlignNet training.

Algorithm 2 Intra-Molecular Alignment Module

Require: A mini-batch of molecules $\mathcal{B}_M = \{M_i = (M_{i,\text{seq}}, M_{i,\text{str}})\}_{i=1}^N$, where M_i can be either a protein or a ligand
Require: Sequence encoder $\text{Enc}_{\text{seq}}(\cdot)$, structure encoder $\text{Enc}_{\text{str}}(\cdot)$, shared Transformer encoder $\text{Transformer}_{\text{shared}}(\cdot)$
Require: Temperature τ
Ensure: Alignment loss $\mathcal{L}_{\text{align}}$ and aligned molecular representations $\{\mathcal{Z}_i^{\text{align}}\}_{i=1}^N$

```
Initialize view collections V^str ← [], V^noise ← [], V^drop ← [], V^seq ← []
Initialize aligned representation collection Z^align ← []
for each molecule M_i in B_M do
    Feature fusion
    E_{i,seq} ← Enc_seq(M_{i,seq})
    E_{i,str} ← Enc_str(M_{i,str})
    Z_{f,i} ← Transformer_shared([E_{i,seq}; E_{i,str}])
    Uni- and multi-modal view generation
    Z_{f,i}^str ← StructuralMask(Z_{f,i})
    Z_{f,i}^noise ← AddRandomNoise(Z_{f,i})
    Z_{f,i}^drop ← EmbeddingDropout(Z_{f,i})
    Z_{f,i}^seq ← SequenceMask(Z_{f,i})
    Append Z_{f,i}^str to V^str
    Append Z_{f,i}^noise to V^noise
    Append Z_{f,i}^drop to V^drop
    Append Z_{f,i}^seq to V^seq
    Append Z_{f,i} to Z^align
end for
Batch-wise uni- and multi-modal alignment objective
L_align ← −I_NCE(V^noise, V^drop; τ)
    − 1/2 [I_NCE(V^str, V^noise; τ) + I_NCE(V^str, V^drop; τ)]
    − 1/2 [I_NCE(V^seq, V^noise; τ) + I_NCE(V^seq, V^drop; τ)]
Update intra-molecular alignment encoder parameters by back-propagating L_align
```

In math notation, step 17:

$$\mathcal{L}_{\text{align}} \leftarrow -I_{\text{NCE}}(\mathcal{V}^{\text{noise}}, \mathcal{V}^{\text{drop}}; \tau) - \frac{1}{2}\left[I_{\text{NCE}}(\mathcal{V}^{\text{str}}, \mathcal{V}^{\text{noise}}; \tau) + I_{\text{NCE}}(\mathcal{V}^{\text{str}}, \mathcal{V}^{\text{drop}}; \tau)\right] - \frac{1}{2}\left[I_{\text{NCE}}(\mathcal{V}^{\text{seq}}, \mathcal{V}^{\text{noise}}; \tau) + I_{\text{NCE}}(\mathcal{V}^{\text{seq}}, \mathcal{V}^{\text{drop}}; \tau)\right]$$

C Interaction-Aware Encoding Module

The complex is modeled as a graph $\mathcal{G} = (\mathcal{V}, \mathcal{E})$ where nodes $\mathcal{V}$ are atoms with initial features $\mathbf{x}_i \in \mathbb{R}^d$ and coordinates $\mathbf{r}_i \in \mathbb{R}^3$. The edges $\mathcal{E}$ represent both intra-molecular covalent bonds and inter-molecular non-covalent interactions within

a 5Å cutoff. The GNN updates the initial node features $\mathbf{x}_i$ by iteratively aggregating information from neighboring nodes, incorporating their features and relative spatial coordinates $\mathbf{r}_i$. This process ensures that the learned representations are invariant to rotation and translation. The updated node hidden state $\mathbf{h}_i$ after several layers of message passing is given by:

$$\mathbf{h}_i = \mathrm{GNN}(\mathbf{x}_i, \mathbf{r}_i, \{\mathbf{x}_j, \mathbf{r}_j\}_{j\in\mathcal{N}(i)}), \tag{1}$$

where $\mathcal{N}(i)$ is the set of neighbors of node v_i. After the final layer of message passing, we obtain the updated node embeddings $\{\mathbf{h}_i\}_{i\in\mathcal{V}}$. To derive graph-level representations for the protein and the ligand, we apply an average pooling operation over their respective sets of nodes, $\mathcal{V}_p$ and $\mathcal{V}_l$:

$$\mathbf{E}_p^{\mathrm{GNN}} = \frac{1}{|\mathcal{V}_p|}\sum_{i\in\mathcal{V}_p}\mathbf{h}_i, \mathbf{E}_l^{\mathrm{GNN}} = \frac{1}{|\mathcal{V}_l|}\sum_{i\in\mathcal{V}_l}\mathbf{h}_i. \tag{2}$$

D Detailed Descriptions of Baseline Methods

D.1 Sequence-based Methods

- **DeepDTA** [1] utilizes two independent CNNs to extract features from SMILES strings and protein sequences, which are then concatenated for affinity prediction.
- **CAPLA** [2] employs the cross-attention mechanism to capture the mutual effect of protein-binding pocket and ligand.
- **GraphDTA** [3] represents ligands as molecular graphs and uses GNNs to capture their structural features more effectively than 1D SMILES strings.
- **MGraphDTA** [4] introduces a multi-scale GNN to capture both local and global structural information of compounds.
- **AttentionSiteDTI** [5] employs a GNN-based architecture to capture relational semantics between protein pockets and ligands, framing the task as a sentence classification problem.
- **DEAttentionDTA** [6] combines dynamic word embeddings with a self-attention mechanism to effectively capture context-dependent features and long-range dependencies in protein sequences.

D.2 Structure-based Methods

- **SchNet** [7] is an equivariant deep learning architecture that uses continuous-filter convolutions to model atomic interactions for quantum chemistry tasks.
- **PotentialNet** [8] is a staged GNN that hierarchically captures structural features by first propagating information in local substructures before integrating global graph-level features.
- **EGNN** [9] is an equivariant GNN that models 3D molecular structures while maintaining equivariance to translations and rotations with high computational efficiency.
- **GAABind** [10] is a geometry-enhanced attention-based model that predicts binding poses and affinities in a multitask framework using dedicated interaction blocks.
- **GVP-GNN** [11] proposes geometric vector perceptrons, which extend standard dense layers to operate directly on Euclidean vectors, enabling GNNs to leverage both geometric and relational information.
- **ProNet** [12] proposes a hierarchical graph network that learns 3D protein representations by capturing the intrinsic hierarchical relationships between different levels of granularity (e.g., amino acid, backbone, all-atom).
- **GET** [13] proposes a Generalist Equivariant Transformer (GET) that universally represents any 3D complex as a geometric graph of sets and uses a bilevel attention module to capture both cross-domain interaction physics and intra-domain hierarchical information.
- **GNN-DTI** [14] models the 3D complex using a GNN with a distance-aware graph attention mechanism to capture inter-atomic interactions.
- **IGN** [15] stacks two separate graph convolution modules to sequentially learn intra- and inter-molecular interactions from the 3D complex structure.

- **GIGN** [16] performs fine-grained interaction modeling by treating covalent and non-covalent interactions as distinct edge types, which are processed separately.
- **CheapNet** [17] employs a hierarchical framework to integrate atom-level and cluster-level interactions, using cross-attention between clusters to focus on relevant interactions.

D.3 Multimodal-based Methods

- **DPLA** [18] combines protein sequence and structural properties with ligand fingerprints and network features through a unified deep learning model.
- **DataDTA** [19] utilizes protein pocket descriptors and sequences alongside ligand SMILES and algebraic graph features, employing a dual-interaction aggregation network to learn from these varied inputs.
- **HaPPy** [20] represents the protein-ligand system through multiple graph-based perspectives and fuses the information from these heterogeneous graphs for binding affinity prediction.
- **MFE** [21] employs a cross-attention strategy to align features from a protein's sequence, 3D structure, and surface to generate a comprehensive protein representation.

D.4 Pretraining-based Methods

- **EGNN-PLM** [22] enhances geometric networks by integrating knowledge from pre-trained protein language models (PLMs), combining the strengths of 1D sequence and 3D structural information.
- **Uni-Mol** [23] is a universal 3D molecular representation learning framework based on an SE(3) Transformer, pre-trained on a massive database of molecular conformations and protein pockets.
- **ProFSA** [24] is a pocket pre-training approach that simulates ligand-receptor interactions by segmenting protein structures and uses contrastive learning to align the pocket with pre-trained small molecule representations.
- **BindNet** [25] is a self-supervised method that learns binding representations by predicting the final complex structure from a given pocket and ligand, using tasks like distance map prediction and masked ligand reconstruction.

D.5 Large-scale Virtual Screening related Methods.

- **NN-score** [26]: A neural-network-based scoring function that predicts a pKd value based on binding characteristics.
- **RFscore** [27]: A scoring function using a Random Forest model to implicitly capture binding effects without explicit physical modeling.
- **Pafnucy** [28]: A 3D CNN model that processes the protein-ligand complex on a 3D grid to estimate binding affinity.

- **OnionNet** [29]: A 3D CNN model that uses element-pair-specific contacts grouped into different distance shells to represent the protein-ligand interaction.
- **Planet** [30]: A multi-task GNN that takes a 3D pocket and 2D ligand graph as input to predict affinity, contact maps, and ligand distance matrices.

E Implementation and Hyperparameter Details

All models were implemented in PyTorch and trained on an Ubuntu server equipped with 256GB of RAM and NVIDIA RTX 4090 (24GB) and NVIDIA RTX A6000 (48GB) GPUs. Model parameters were initialized using Xavier initialization and optimized with the Adam optimizer. We performed a comprehensive hyperparameter search for both the intra-molecular alignment pre-training and the binding affinity prediction task. The search spaces and the final optimal hyperparameters are detailed in Table S1 and Table S2.

Table S1: Hyperparameter search space of intra-molecular alignment pre-training for protein and ligand. The optimal hyperparameters are shown in **bold**.

**Hyperparameter**	**Protein**	**Ligand**
Sequence Encoder	**ESM-2** [31]	**MolFormer** [32]
Structure Encoder	ESM-IF1 [31]<br>**GearNet** [33]	Uni-Mol [23]<br>**GraphMVP** [34]
Batch Size	16, **32**	16, 32, **64**
Transformer Layers	2, **4**, 8	2, **4**, 8
Transformer Attention Heads	2, **4**, 8	2, **4**, 8
Hidden Dimension	768, **1024**, 2048	512, **768**, 1024
Learning Rate	1e-4, **5e-5**, 1e-5, 1e-6	**5e-5**, 1e-5, 1e-6
Epochs	**100**	**100**

Table S2: Hyperparameter search space for the binding affinity prediction tasks. The optimal hyperparameters are shown in **bold**.

**Hyperparameter**	**Cross-dataset**	**LBA 30%**	**LBA 60%**
Learning Rate	1e-4, **5e-5**, 1e-5	**5e-5**	**5e-5**
Loss Weight (α^a)	$--^c$	**0.5**	**0.3**
Loss Weight (β^b)	$--^c$	**0.5**	**0.7**
Batch Size	16, **32**, 64	16, **32**	16, **32**
Epochs	**40**	**20**	**20**

a: the search space of α is [0.1, 0.9], limited by space, we only show the optimal hyperparameters
b: the search space of β is [0.1, 0.9], limited by space, we only show the optimal hyperparameters
c: For the cross-dataset generalization task, due to the large data volume, we employed the uncertainty-based method proposed by [35] to automatically learn the weights for the loss components instead of performing a grid search

F Detailed Performance with Standard Deviations and Statistical Analysis.

Following the evaluation protocol of CheapNet[17], all experiments were conducted three times using different random seeds. This section presents the full results for the binding affinity prediction tasks, reported as the mean ± standard deviation. We also performed an independent samples t-test to assess the statistical significance of the improvement of our full AlignNet model over the strongest baseline, CheapNet, as shown in Tables Table S3 and Table S4. Although the

improvements in the RMSE metric are not strictly statistically significant ($p > 0.05$), AlignNet demonstrates statistically significant gains in the correlation-based metrics (PCC and SCC) across all datasets. This strong performance in correlation highlights that AlignNet is highly suitable for ranking-related tasks, which are crucial for large-scale virtual screening in real-world drug discovery applications.

Table S3: Full performance comparison on the cross-dataset generalization task.[a]

**Model**	**v2016 core set**		**v2019 holdout set**	
	**RMSE ↓**	**PCC ↑**	**RMSE ↓**	**PCC ↑**
**Sequence-based**				
DeepDTA[b]	1.357 ± 0.015	0.785 ± 0.007	1.485 ± 0.023	0.586 ± 0.012
GraphDTA[b]	1.434 ± 0.064	0.754 ± 0.025	1.705 ± 0.075	0.474 ± 0.028
MGraphDTA[b]	1.439 ± 0.047	0.753 ± 0.022	1.553 ± 0.028	0.538 ± 0.013
AttentionSiteDTI[b]	1.352 ± 0.022	0.784 ± 0.008	1.539 ± 0.015	0.563 ± 0.004
**Structure-based**				
PotentialNet[b]	1.503 ± 0.033	0.772 ± 0.007	1.514 ± 0.028	0.564 ± 0.014
SchNet[b]	1.390 ± 0.023	0.787 ± 0.016	1.522 ± 0.071	0.560 ± 0.028
EGNN[b]	1.289 ± 0.021	0.816 ± 0.011	1.399 ± 0.013	0.628 ± 0.010
GNN-DTI[b]	1.384 ± 0.013	0.779 ± 0.008	1.446 ± 0.006	0.614 ± 0.007
IGN[b]	1.269 ± 0.030	0.821 ± 0.013	1.410 ± 0.015	0.630 ± 0.008
GIGN[b]	1.190 ± 0.017	0.840 ± 0.007	1.393 ± 0.007	0.641 ± 0.006
CheapNet[b]	**1.107 ± 0.011**	0.870 ± 0.002	1.343 ± 0.007	0.665 ± 0.003
**Multi-modal-based**				
HaPPy[c]	1.213 ± 0.025	0.823 ± 0.014	-	-
MFE[c]	<u>1.150 ± 0.023</u>	0.845 ± 0.005	-	-
**Ours**				
$\text{AlignNet}_{\text{seq}}$	1.343 ± 0.064	<u>0.969 ± 0.008</u>	1.203 ± 0.035	0.945 ± 0.009
$\text{AlignNet}_{\text{ss}}$	1.264 ± 0.049	0.967 ± 0.009	<u>1.159 ± 0.025</u>	<u>0.947 ± 0.008</u>
$\text{AlignNet}_{\text{full}}$	1.213 ± 0.073	**0.971 ± 0.006**	**1.078 ± 0.080**	**0.963 ± 0.002**
p-value[d]	1.257 × 1e-1	6.263 × 1e-4	2.846 × 1e-2	4.518 × 1e-8

a: The top results are shown in **bold**, and the second-best are <u>underlined</u>, respectively. The dash sign indicates the results of the corresponding methods are not available.
b: Results are from CheapNet [17].
c: Results are from MFE [21].
d: The reported p-values are derived from an independent samples t-test comparing $\text{Align}_{\text{full}}$ against the strongest baseline, CheapNet.

Table S4: Full performance comparison on the diverse protein generalization task.[a]

Model	LBA 30%			LBA 60%		
	**RMSE ↓**	**PCC ↑**	**SCC ↑**	**RMSE ↓**	**PCC ↑**	**SCC ↑**
**Sequence-based**						
DeepDTA[b]	1.866 ± 0.080	0.472 ± 0.022	0.471 ± 0.024	1.762 ± 0.261	0.666 ± 0.012	0.663 ± 0.015
Atom3D-ENN[b]	1.985 ± 0.006	0.165 ± 0.006	0.152 ± 0.024	1.891 ± 0.004	0.249 ± 0.006	0.275 ± 0.008
TAPE[b]	1.890 ± 0.035	0.338 ± 0.044	0.286 ± 0.124	1.633 ± 0.016	0.568 ± 0.033	0.571 ± 0.021
**Structure-based**						
Atom3D-GNN[b]	1.601 ± 0.048	0.545 ± 0.027	0.533 ± 0.033	1.408 ± 0.069	0.743 ± 0.022	0.743 ± 0.027
IEConv[b]	1.554 ± 0.016	0.414 ± 0.053	0.428 ± 0.032	1.473 ± 0.024	0.667 ± 0.011	0.675 ± 0.019
ProNet[b]	1.463 ± 0.001	0.551 ± 0.005	0.551 ± 0.008	1.343 ± 0.025	0.765 ± 0.009	0.761 ± 0.003
SchNet[b]	1.370 ± 0.028	0.590 ± 0.017	0.571 ± 0.028	-	-	-
LEFTNet[b]	1.366 ± 0.016	0.592 ± 0.014	0.580 ± 0.011	-	-	-
GET[b]	1.327 ± 0.005	0.620 ± 0.004	0.611 ± 0.003	-	-	-
CheapNet[b]	<u>1.311 ± 0.003</u>	0.642 ± 0.001	0.639 ± 0.010	1.238 ± 0.005	0.794 ± 0.002	0.789 ± 0.001
**Pre-training-based**						
EGNN-PLM[b]	1.403 ± 0.010	0.565 ± 0.020	0.544 ± 0.010	1.559 ± 0.020	0.644 ± 0.020	0.646 ± 0.020
ProFSA[b]	1.377 ± 0.010	0.628 ± 0.010	0.620 ± 0.010	1.377 ± 0.010	0.764 ± 0.000	0.762 ± 0.010
**Multi-modal-based**						
HaPPy[c]	1.814 ± 0.034	0.612 ± 0.010	0.606 ± 0.012	2.179 ± 0.025	0.699 ± 0.005	0.698 ± 0.006
MFE[c]	1.941 ± 0.137	0.598 ± 0.006	0.594 ± 0.001	1.813 ±0.036	0.759 ± 0.001	0.763 ± 0.001
**Ours**						
$\text{AlignNet}_{\text{seq}}$	1.464 ± 0.022	0.827 ± 0.020	0.843 ± 0.027	<u>1.221 ± 0.174</u>	0.919 ± 0.009	0.952 ± 0.005
$\text{AlignNet}_{\text{ss}}$	1.465 ± 0.015	<u>0.846 ± 0.013</u>	<u>0.847 ± 0.010</u>	1.227 ± 0.245	<u>0.921 ± 0.010</u>	<u>0.952 ± 0.005</u>
**$\text{AlignNet}_{\text{full}}$**	**1.301 ± 0.065**	**0.868 ± 0.020**	**0.881 ± 0.021**	**1.056 ± 0.155**	**0.922 ± 0.006**	**0.956 ± 0.003**
p-value[d]	8.256 × 1e-1	2.600 × 1e-3	3.406 × 1e-4	1.804 × 1e-1	1.035 × 1e-4	3.021 × 1e-5

a: The top results are shown in **bold**, and the second-best are <u>underlined</u>, respectively. The dash sign indicates the results of the corresponding methods are not available.
b: Results are from CheapNet [17].
c: Results are from source code.
d: The reported p-values are derived from an independent samples t-test comparing $\text{Align}_{\text{full}}$ against the strongest baseline, CheapNet.

G Mutant-Protein dataset

In this section, we show the dataset of Mutant-Protien generalization task (Table S5).

Table S5: The PDB_ID for Mutant-Protien generalization task.

2IEO	2IDW	3CYW	3D1Z	3D20	3LZU	2QCI	2QD6	2QD8	2QD7	3UM6	2PYN
2Q63	2PYM	2IPW	2AOC	3LQG	3LEP	3M4H	2PDU	2PDC	2PDJ	2PDP	2VFG
2VFE	3NU5	3NU6	3NUJ	3NU9	3NUO	3NU4	2PDQ	2PDH	2PDN	3N0S	3N0M
1GZ3	3G0F	3RDO	2OGY	3GUG	2WDS	3UM5	1J3J	1SYI	1NJA	1NJC	3F8Y
3F91	3F8Z	1YKL	1W6Y	1QKT	2PDW	2PDY	2PD9	3A6Q	1OBO	1OBV	2YGE
2YGF	1Q6E	1Q6D	1Q6G	2QFT	2F8F	1YDK	2ITO	3LQL	3LBO	3LD5	3LZ5
1SDU	1SDV	1U70	1DLS	1N8K	3RHQ	3RHJ	3RHO	3RHR	3M3E	3M3O	1OVD
1JQV	1JRC	1JRB	1JQX	3MOF	2ISF	3UJC	3UJD	1MGO	3MOH	1QDS	1S89
1TPW	1YP6	3CYX	3D1X	3D1Y	2NNP	2NMZ	2QFS	3FJX	3FK0	3MOE	1HK2
1HK3	1HK5	3AM5	1E2L	2ONZ	2PDL	3FRB	3N0H	3S3V	1A9M	1JMF	1JMG
1JMH	1JMI	1TSV	1TSY	1J3K	3FY9	3FY8	1BWA	2PDB	2PDI	2PDF	2PDM
2PDX	2PD5										

H Detailed of COVID Moonshot dataset

The COVID Moonshot data used in the Cross-Docking Generalization experiment are derived from the high-throughput SARS-CoV-2 MPro crystallographic and biochemical dataset reported by [36], which contains hundreds of MPro–ligand crystal structures with associated activity measurements. In the original study, docked structures were generated by redocking COVID Moonshot compounds against the Diamond/XChem MPro-x2908 structure using OpenEye's "Classic OEDocking" floe v0.7.2 on the Orion 2020.3.1 Academic Stack; ligand conformers and stereochemistry were enumerated by Omega with up to 500 conformations, and docking was performed with FRED in HYBRID mode using the x2908 bound ligand as the reference.

For the Cross-Docking Generalization benchmark, we followed the GAABind protocol[10]. Specifically, the origin dataset subset contained 199 MPro–ligand complexes; three ligands that could not be processed by RDKit were removed, resulting in 196 ligands. These ligands were cross-docked to a single non-cognate MPro holo receptor, x11428, which was selected as the highest-resolution protein structure in the dataset. The binding pocket was defined as residues containing atoms within 6 Å of any ligand heavy atom. For each ligand, RDKit ETKDG was used to generate 100 apo conformations, which were clustered by KMeans into 10 representative conformations. During evaluation, predictions were made over these conformations, and the final pose was selected according to the lowest generation loss; for conventional docking baselines, multiple runs were performed and the top-scoring pose was selected.

I Large-scale Virtual Screening Dataset

In this section, we show the non-overlapping DUD-E subset used for zero-shot large-scale virtual screening (Table S6).

Table S6: Characterization of the non-overlapping DUD-E subset used for zero-shot validation, detailing the number of active and decoy molecules per target.

**Target**	**Actives**	**Decoys**	**Target**	**Actives**	**Decoys**	**Target**	**Actives**	**Decoys**
HIVPRT	1395	36278	AOFB	168	6931	HXK4	127	4803
CXCR	162	7707	PPARD	288	13232	DHI1	519	19623
SAHH	190	3483	ADRB2	447	15255	BACE1	485	18221
PGH2	531	23405	FA10	792	20417	ESR2	595	20313
GRIA2	297	12061	DPP4	1079	41372	ADRB1	458	15958
TYSY	311	6883	FKB1A	273	5832	CP3A4	363	11940
INHA	71	2318	ACES	664	26373	PGH1	251	10942
CASP3	350	10822	ALDR	220	9136	GLCM	313	3837
TRY1	758	26219	KITH	132	2866	THRB	861	27321
GCR	563	15184	FA7	185	6302	TRYB1	171	7713
COMT	86	3926	PPARA	544	19831	PYRD	134	6648
GRIK1	152	6617	UROK	306	9933	DYX	566	17384
FPPS	213	9015	DRD3	877	34188	HIVRT	639	19134

J Performance Evaluation on Davis dataset

We adopted the dataset from the FDA framework [37], where protein-ligand complexes were constructed by first predicting protein structures from amino acid sequences using ColabFold [38], followed by ligand docking with DiffDock [39]. The dataset contains 68 ligands and 442 proteins, comprising 14,425 protein-ligand binding affinity measurements. For robust evaluation, we assessed model performance under the unseen-ligand and unseen-both settings. In the unseen-ligand split, 20% of the unique drugs were held out for the test set based on molecular scaffolds, simulating a real-world drug discovery scenario where the model must predict binding affinities for novel chemical entities. In the more challenging unseen-both split, 20% of the unique proteins and 20% of the unique drugs were simultaneously held out, ensuring that the test set consists entirely of protein-ligand pairs where neither entity was exposed to the model during training. Following the evaluation protocol, we evaluated models under five strictly aligned train/test splits and reported the mean and standard deviation as the final results. MSE and PCC were used as evaluation metrics.

As shown in Table S7, AlignNet achieves consistent and robust improvements on the Davis dataset under both the unseen-both and unseen-ligand settings. In the highly challenging unseen-both scenario, $\text{Align}_{\text{full}}$ significantly outperforms

all baselines in predicting affinity rankings, improving PCC by 24.1% over the strongest baseline, while reducing MSE by 4.2%. Under the unseen-ligand setting, $\text{Align}_{\text{full}}$ ranks first across both metrics, delivering a 3.7% reduction in MSE and a 11.9% improvement in PCC compared to the best-performing baseline. Notably, when compared directly to the FDA model, $\text{Align}_{\text{full}}$ achieves MSE reductions of 4.2% and 12.6%, alongside PCC improvements of 24.1% and 11.9% in the unseen-both and unseen-ligand settings, respectively. Beyond these accuracy gains, all three AlignNet variants exhibit exceptionally small standard deviations across splits, in stark contrast to the large variability seen in the baselines. These results confirm that AlignNet provides superior stability and robust generalization for out-of-distribution drug discovery tasks.

Table S7: Comparison of affinity prediction performance on DAVIS under unseen-both and unseen-ligand settings. [a]

**Method**	**Unseen-both**		**Unseen-ligand**	
	**MSE↓**	**PCC↑**	**MSE↓**	**PCC↑**
DeepDTA[1] [b]	0.631±0.075	0.153±0.032	0.967±0.053	0.234±0.025
GraphDTA[3] [b]	0.664±0.071	0.043±0.027	1.026±0.195	0.315±0.087
DGraphDTA[40] [b]	0.609±0.040	0.145±0.003	0.943±0.016	0.266±0.030
MGraphDTA[4] [b]	0.661±0.191	0.225±0.027	0.955±0.034	0.277±0.015
FDA[37] [b]	0.570±0.035	0.303±0.038	1.039±0.178	0.320±0.032
AlignNet_{seq}	0.563±0.003	0.321±0.003	<u>0.932±0.037</u>	0.334±0.041
AlignNet_{ss}	<u>0.559±0.002</u>	<u>0.345±0.002</u>	0.936±0.039	<u>0.340±0.032</u>
AlignNet_{full}	**0.546±0.005**	**0.376±0.006**	**0.908±0.057**	**0.358±0.034**

[a:] The top results are shown in **bold**, and the second-best are <u>underlined</u>, respectively.
[b:] Results are from source code.

K Large-scale Virtual Screening

Large-scale virtual screening aims to prioritize active compounds from large compound libraries. Beyond overall ranking performance, its practical utility depends heavily on the early recognition of active compounds near the top of the ranked list. We evaluated AlignNet on a virtual screening task using the widely adopted DUD-E benchmark [41], following the protocol of Planet [30] to ensure that no protein targets overlapped with our training set. After preprocessing, 39 processed targets were retained, as provided in Appendix Section I. We used AUROC to assess the overall ability to rank active compounds above decoys across the entire dataset. To evaluate early enrichment, we further reported BEDROC, which assigns greater weight to active compounds ranked near the top, as well as enrichment factors (EFs) at the top 0.5%, 1%, and 5% thresholds. The baseline methods mainly include virtual screening models such as Planet [30]. Since 3D protein–ligand complex structures are often unavailable in practical virtual screening scenarios, we evaluated the complex-free variants $\text{AlignNet}_{\text{ss}}$ and $\text{AlignNet}_{\text{seq}}$.

As shown in Table S8, AlignNet achieves competitive performance on the DUD-E virtual screening benchmark. Even the sequence-only variant, AlignNet∗seq, outperforms the structure-based Planet model in AUROC and achieves a higher BEDROC than all structure-based baselines that report this metric. With the incorporation of structural information, AlignNet∗ss further improves performance, achieving the best BEDROC and the highest EF values at the top 0.5%, 1%, and 5% thresholds. Although AlignNetfull obtains the highest AUROC, it does not achieve the best performance across all early-enrichment metrics. This trade-off likely occurs because the fine-grained 3D representations in $\text{AlignNet}_{\text{full}}$ are highly sensitive to docking inaccuracies and structural noise; thus, slight pose deviations can penalize true actives at the very top of the ranking. In contrast, $\text{AlignNet}_{\text{ss}}$ relies on more robust structural features that are less affected by such domain shifts, delivering the strongest early-enrichment performance. These results suggest that $\text{AlignNet}_{\text{ss}}$ provides a flexible and practically useful solution for virtual screening scenarios where experimentally resolved or reliably predicted complex structures are difficult to obtain.

Table S8: Performance comparison on the large-scale virtual screening task. [a]

Model	AUROC↑ (%)	BEDROC↑ (%)	EF↑		
			0.5%	1%	5%
NN-score[26] [b]	68.30	12.20	4.16	4.02	3.12
RFscore[27] [b]	65.21	12.41	4.90	4.52	2.98
Pafnucy[28] [b]	63.11	16.50	4.24	3.86	3.76
OnionNet[29] [b]	59.71	8.62	2.84	2.84	2.20
Planet[30] [b]	71.60	-	<u>10.23</u>	8.83	5.40
$\text{AlignNet}_{\text{seq}}$	74.31	22.11	9.31	7.15	5.17
$\text{AlignNet}_{\text{ss}}$	<u>76.34</u>	**28.53**	**10.26**	**9.62**	**6.38**
$\text{AlignNet}_{\text{full}}$	**76.70**	<u>27.08</u>	9.88	<u>9.12</u>	<u>6.14</u>

[a]: The top results are shown in **bold**, and the second-best are <u>underlined</u>, respectively. The dash sign indicates the results of the corresponding methods are not available.
[b]: Results are from Planet[30].

References

1. Hakime Öztürk, Arzucan Özgür, and Elif Ozkirimli. Deepdta: deep drug–target binding affinity prediction. *Bioinformatics*, 34(17):i821–i829, 2018.
2. Zhi Jin, Tingfang Wu, Taoning Chen, Deng Pan, Xuejiao Wang, Jingxin Xie, Lijun Quan, and Qiang Lyu. Capla: improved prediction of protein–ligand binding affinity by a deep learning approach based on a cross-attention mechanism. *Bioinformatics*, 39(2):btad049, 2023.
3. Thin Nguyen, Hang Le, Thomas P Quinn, Tri Nguyen, Thuc Duy Le, and Svetha Venkatesh. Graphdta: predicting drug–target binding affinity with graph neural networks. *Bioinformatics*, 37(8):1140–1147, 2021.
4. Ziduo Yang, Weihe Zhong, Lu Zhao, and Calvin Yu-Chian Chen. Mgraphdta: deep multiscale graph neural network for explainable drug–target binding affinity prediction. *Chemical science*, 13(3):816–833, 2022.
5. Mehdi Yazdani-Jahromi, Niloofar Yousefi, Aida Tayebi, Elayaraja Kolanthai, Craig J Neal, Sudipta Seal, and Ozlem Ozmen Garibay. Attentionsitedti: an interpretable graph-based model for drug-target interaction prediction using nlp sentence-level relation classification. *Briefings in Bioinformatics*, 23(4):bbac272, 2022.
6. Xiying Chen, Jinsha Huang, Tianqiao Shen, Houjin Zhang, Li Xu, Min Yang, Xiaoman Xie, Yunjun Yan, and Jinyong Yan. Deattentiondta: protein–ligand binding affinity prediction based on dynamic embedding and self-attention. *Bioinformatics*, 40(6):btae319, 2024.
7. Kristof Schütt, Pieter-Jan Kindermans, Huziel Enoc Sauceda Felix, Stefan Chmiela, Alexandre Tkatchenko, and Klaus-Robert Müller. Schnet: A continuous-filter convolutional neural network for modeling quantum interactions. *Advances in neural information processing systems*, 30, 2017.
8. Evan N Feinberg, Debnil Sur, Zhenqin Wu, Brooke E Husic, Huanghao Mai, Yang Li, Saisai Sun, Jianyi Yang, Bharath Ramsundar, and Vijay S Pande. Potentialnet for molecular property prediction. *ACS central science*, 4(11):1520–1530, 2018.
9. Vıctor Garcia Satorras, Emiel Hoogeboom, and Max Welling. E (n) equivariant graph neural networks. In *International conference on machine learning*, pages 9323–9332. PMLR, 2021.
10. Huishuang Tan, Zhixin Wang, and Guang Hu. Gaabind: a geometry-aware attention-based network for accurate protein–ligand binding pose and binding affinity prediction. *Briefings in Bioinformatics*, 25(1):bbad462, 2024.
11. Bowen Jing, Stephan Eismann, Patricia Suriana, Raphael John Lamarre Townshend, and Ron Dror. Learning from protein structure with geometric vector perceptrons. In *International Conference on Learning Representations*, 2021.
12. Limei Wang, Haoran Liu, Yi Liu, Jerry Kurtin, and Shuiwang Ji. Learning hierarchical protein representations via complete 3d graph networks. In *The Eleventh International Conference on Learning Representations*, 2023.
13. Xiangzhe Kong, Wenbing Huang, and Yang Liu. Generalist equivariant transformer towards 3d molecular interaction learning, 2024.
14. Jaechang Lim, Seongok Ryu, Kyubyong Park, Yo Joong Choe, Jiyeon Ham, and Woo Youn Kim. Predicting drug–target interaction using a novel graph neural network with 3d structure-embedded graph representation. *Journal of chemical information and modeling*, 59(9):3981–3988, 2019.
15. Dejun Jiang, Chang-Yu Hsieh, Zhenxing Wu, Yu Kang, Jike Wang, Ercheng Wang, Ben Liao, Chao Shen, Lei Xu, Jian Wu, et al. Interactiongraphnet: a novel and

efficient deep graph representation learning framework for accurate protein–ligand interaction predictions. *Journal of medicinal chemistry*, 64(24):18209–18232, 2021.
16. Ziduo Yang, Weihe Zhong, Qiujie Lv, Tiejun Dong, and Calvin Yu-Chian Chen. Geometric interaction graph neural network for predicting protein–ligand binding affinities from 3d structures (gign). *The journal of physical chemistry letters*, 14(8):2020–2033, 2023.
17. Hyukjun Lim, Sun Kim, and Sangseon Lee. Cheapnet: Cross-attention on hierarchical representations for efficient protein-ligand binding affinity prediction. In *The Thirteenth International Conference on Learning Representations*, 2025.
18. Wei Wang, Bin Sun, Dong Liu, Xianfang Wang, and Hongjun Zhang. Dpla: prediction of protein-ligand binding affinity by integrating multi-level information. In *2021 IEEE international conference on bioinformatics and biomedicine (BIBM)*, pages 3428–3434. IEEE, 2021.
19. Yan Zhu, Lingling Zhao, Naifeng Wen, Junjie Wang, and Chunyu Wang. Datadta: a multi-feature and dual-interaction aggregation framework for drug–target binding affinity prediction. *Bioinformatics*, 39(9):btad560, 2023.
20. Xianfeng Zhang, Yanhui Gu, Guandong Xu, Yafei Li, Jinlan Wang, and Zhenglu Yang. Happy: harnessing the wisdom from multi-perspective graphs for protein-ligand binding affinity prediction (student abstract). In *Proceedings of the AAAI Conference on Artificial Intelligence*, volume 37, pages 16384–16385, 2023.
21. Shiyu Xu, Lian Shen, Menglong Zhang, Changzhi Jiang, Xinyi Zhang, Yanni Xu, Juan Liu, and Xiangrong Liu. Surface-based multimodal protein–ligand binding affinity prediction. *Bioinformatics*, 40(7):btae413, 2024.
22. Fang Wu, Yu Tao, Dragomir Radev, and Jinbo Xu. When geometric deep learning meets pretrained protein language models. *bioRxiv*, pages 2023–01, 2023.
23. Gengmo Zhou, Zhifeng Gao, Qiankun Ding, Hang Zheng, Hongteng Xu, Zhewei Wei, Linfeng Zhang, and Guolin Ke. Uni-mol: A universal 3d molecular representation learning framework. In *The Eleventh International Conference on Learning Representations*, 2023.
24. Bowen Gao, Yinjun Jia, YuanLe Mo, Yuyan Ni, Wei-Ying Ma, Zhi-Ming Ma, and Yanyan Lan. Self-supervised pocket pretraining via protein fragment-surroundings alignment. In *The Twelfth International Conference on Learning Representations*, 2024.
25. Shikun Feng, Minghao Li, Yinjun Jia, Wei-Ying Ma, and Yanyan Lan. Protein-ligand binding representation learning from fine-grained interactions. In *The Twelfth International Conference on Learning Representations*, 2024.
26. Jacob D Durrant and J Andrew McCammon. Nnscore 2.0: a neural-network receptor–ligand scoring function. *Journal of chemical information and modeling*, 51(11):2897–2903, 2011.
27. Pedro J Ballester and John BO Mitchell. A machine learning approach to predicting protein–ligand binding affinity with applications to molecular docking. *Bioinformatics*, 26(9):1169–1175, 2010.
28. Marta M Stepniewska-Dziubinska et al. Pafnucy-a deep neural network for structure-based drug discovery. *Bioinformatics*, 1050:19, 2017.
29. Liangzhen Zheng, Jingrong Fan, and Yuguang Mu. Onionnet: a multiple-layer intermolecular-contact-based convolutional neural network for protein–ligand binding affinity prediction. *ACS omega*, 4(14):15956–15965, 2019.
30. Xiangying Zhang, Haotian Gao, Haojie Wang, Zhihang Chen, Zhe Zhang, Xinchong Chen, Yan Li, Yifei Qi, and Renxiao Wang. Planet: a multi-objective graph neural network model for protein–ligand binding affinity prediction. *Journal of Chemical Information and Modeling*, 64(7):2205–2220, 2023.

31. Zeming Lin, Halil Akin, Roshan Rao, Brian Hie, Zhongkai Zhu, Wenting Lu, Nikita Smetanin, Robert Verkuil, Ori Kabeli, Yaniv Shmueli, et al. Evolutionary-scale prediction of atomic-level protein structure with a language model. *Science*, 379(6637):1123–1130, 2023.
32. Jerret Ross, Brian Belgodere, Vijil Chenthamarakshan, Inkit Padhi, Youssef Mroueh, and Payel Das. Large-scale chemical language representations capture molecular structure and properties. *Nature Machine Intelligence*, 4(12):1256–1264, 2022.
33. Zuobai Zhang, Minghao Xu, Arian Rokkum Jamasb, Vijil Chenthamarakshan, Aurelie Lozano, Payel Das, and Jian Tang. Protein representation learning by geometric structure pretraining. In *The Eleventh International Conference on Learning Representations*, 2023.
34. Shengchao Liu, Hanchen Wang, Weiyang Liu, Joan Lasenby, Hongyu Guo, and Jian Tang. Pre-training molecular graph representation with 3d geometry. In *International Conference on Learning Representations*, 2022.
35. Alex Kendall, Yarin Gal, and Roberto Cipolla. Multi-task learning using uncertainty to weigh losses for scene geometry and semantics. In *Proceedings of the IEEE conference on computer vision and pattern recognition*, pages 7482–7491, 2018.
36. Kadi L Saar, William McCorkindale, Daren Fearon, Melissa Boby, Haim Barr, Amir Ben-Shmuel, COVID Moonshot Consortium, Nir London, Frank von Delft, John D Chodera, et al. Turning high-throughput structural biology into predictive inhibitor design. *Proceedings of the National Academy of Sciences*, 120(11):e2214168120, 2023.
37. Ming-Hsiu Wu, Ziqian Xie, and Degui Zhi. A folding-docking-affinity framework for protein-ligand binding affinity prediction. *Communications Chemistry*, 8(1):108, 2025.
38. Milot Mirdita, Konstantin Schütze, Yoshitaka Moriwaki, Lim Heo, Sergey Ovchinnikov, and Martin Steinegger. Colabfold: making protein folding accessible to all. *Nature methods*, 19(6):679–682, 2022.
39. Gabriele Corso, Hannes Stärk, Bowen Jing, Regina Barzilay, and Tommi S. Jaakkola. Diffdock: Diffusion steps, twists, and turns for molecular docking. In *The Eleventh International Conference on Learning Representations*, 2023.
40. Mingjian Jiang, Zhen Li, Shugang Zhang, Shuang Wang, Xiaofeng Wang, Qing Yuan, and Zhiqiang Wei. Drug–target affinity prediction using graph neural network and contact maps. *RSC advances*, 10(35):20701–20712, 2020.
41. Michael M Mysinger, Michael Carchia, John J Irwin, and Brian K Shoichet. Directory of useful decoys, enhanced (dud-e): better ligands and decoys for better benchmarking. *Journal of medicinal chemistry*, 55(14):6582–6594, 2012.
